# Low-Velocity Impact Response of Novel TPMS and Stochastic Lattice Cores of Sandwich Structures

**DOI:** 10.3390/ma18122889

**Published:** 2025-06-18

**Authors:** Alexandru Vasile, Dan Mihai Constantinescu, Iulian Constantin Coropețchi, Ștefan Sorohan, Andrei Ioan Indreș

**Affiliations:** 1 Department of Strength of Materials, National University for Science and Technology POLITEHNICA Bucharest, Splaiul Independeţei 313, 060042 Bucharest, Romania; alexandru.vasile@mta.ro (A.V.); iulian.coropetchi@mta.ro (I.C.C.); stefan.sorohan@upb.ro (Ș.S.); 2 Faculty of Aircraft and Military Vehicles, Military Technical Academy “Ferdinand I”, Boulevard George Coşbuc 39-49, 050141 Bucharest, Romania; andrei.indres@mta.ro; 3 Institute of Solid Mechanics of the Romanian Academy, Str. Constantin Mille No. 15, 010141 Bucharest, Romania

**Keywords:** TPMS, stochastic, implicit modeling, impact testing, sandwich structure

## Abstract

This study explores the mechanical performance of triply periodic minimal surface (TPMS) and stochastic lattice structures subjected to low-velocity impact. Two structurally promising geometries—one TPMS-based and one stochastic—were tested and compared with the well-established gyroid. Specimens were fabricated using stereolithography (SLA) and subjected to impact energies of 30 J and 40 J to assess the structural response and energy absorption capabilities. Experimental results show that the proposed TPMS structure exhibits higher impact forces compared with the gyroid, which are associated with significant impactor displacement and deep indentation. These samples demonstrated extensive damage, with cracking propagating through the entire core at higher energies, highlighting their susceptibility to structural failure despite their high initial strength. On the contrary, the stochastic structures allowed localized deformation in the impacted region, thus successfully avoiding catastrophic failure. The impact force efficiency was higher for both gyroid and stochastic geometries, with values ranging between 0.6 and 0.7, indicating effective energy absorption with reduced internal stress gradients. Furthermore, the evaluation of damping performance showed that most structures displayed high damping, as minimal energy was transferred back to the impactor. This work highlights the feasibility and functional versatility of TPMS and stochastic geometries for use in impact mitigation, vibration control, and related engineering applications.

## 1. Introduction

Low-velocity impact testing is used in engineering to simulate and evaluate the response of materials and structures to impact events that occur at relatively low speeds, typically below 10 m/s [1,2]. This type of testing is essential for applications where structures are exposed to impact events, such as falling objects, collisions with other elements, or accidental impacts. Although these events generally do not cause structural damage, they can induce internal defects that compromise the structural integrity. Evaluating the low-velocity impact response of sandwich configurations with cores formed from TPMS geometries is crucial for understanding their behavior under such conditions and comparing their performance with traditional cores [3].

Experimental procedures for impact testing typically involve the controlled dropping of a mass or impactor onto the surface of structural components, measuring variables such as impact force, energy absorption, and crack propagation. Such tests provide insight into the energy-absorbing capabilities of a structure and the reduction of damage that can occur, key properties for applications requiring structural safety. Unlike traditional foam or honeycomb cores, TPMS structures exhibit distinct deformation mechanisms, including local buckling or progressive densification, which contribute to the improvement of their energy-absorbing capabilities.

A review of the current state of the art in the dynamic response of sandwich structures subjected to low-velocity impacts is presented in refs. [4,5]. Details on the factors influencing the mechanical response of composite sandwich panels, the deformation mechanisms, and the energy absorption efficiency are provided.

Separately, the response to low-velocity impact loading on sandwich structures with specific cores has been studied in various publications in the relevant literature. Boonkong et al. in [6] address curvilinear metal cores subjected to different impact energies, observing the deformation localization, internal wall buckling, and top-face rupture at an energy of 80 J. Primitive TPMS structures and one stochastic geometry, made of SS316L stainless steel, with a relative density of 28.1%, are studied in [7]. The results suggest that the Primitive structure offered better puncture resistance than the stochastic counterpart, while it achieved a better distribution of stresses in the mass of the part, leading to higher values of deformation that help internal dissipation of the impact energy. The influence that the relative density and cell dimensions have on the maximum impact load is also presented. Fashanu et al. [8] use three types of cores (hexagonal, gyroid, and Schwartz D) made out of steel powders, which are tested with an impact energy of 9.3 J. The results show that both TPMS topologies offer superior results to the conventional hexagonal structure. Regarding the maximum recorded load, the gyroid achieved 28% higher values, and the increase was 39% for the Schwartz D geometry. The absorbed energy was also 9% higher in the case of the gyroid and 16% in the case of the Schwartz D structure. Therefore, both TPMS topologies offer good potential as alternatives to the honeycomb structure, especially in applications where improved energy absorption is required. The impact responses at various energy levels for re-entrant and honeycomb geometries made of Polylactic Acid (PLA) are compared by Özen et al. [9]. The results show that sandwich panels with re-entrant cores demonstrate superior impact resistance and better energy dissipation compared to those with honeycomb cores. Bates et al. [10] studied the impact of using a simple hexagonal configuration and a thickness gradient in height, made of Thermoplastic Polyurethane (TPU). One of the conclusions suggests that the gradient structures are more effective in absorbing low (36 mJ/cm^3^) and high (270 mJ/cm^3^) impact energies, while the uniform density structures perform better in absorbing intermediate impact energies (143 mJ/cm^3^). Almesmari et al. [11] evaluated the response to impact loads of different energies of cubic geometry (BCC) and a custom geometry, made by fused deposition modeling (FDM) of Acrylonitrile Butadiene Styrene (ABS). Although the new proposed designs did not reach the same maximum transmitted force value, they offered superior performances in terms of puncture limit and impact energy absorption efficiency.

The effects of filler addition have been widely studied in multiple publications. Hao et al. [12] investigate a sandwich structure featuring a hexagonal core, which was injected with vulcanized silicone. Impact tests with an energy of 25 J showed an improvement in the energy absorption capacity and a reduction in the impact force fluctuations. The filling of a cubic lattice structure made of PLA with polyurethane foams is proposed in [13] and tested at a speed of 2.12 m/s and an impact energy of 3.14 J. The conclusions indicate that to minimize shock impact, the foams should possess a low elastic modulus to avoid altering the part’s structural rigidity. The viscosity of the foams can help to dissipate more energy and stabilize the initiation and propagation of cracks. Also, high ductility leads to improved energy absorption, allowing significant deformation during impact. Karahan et al. [14] propose a proprietary topology made of a resin and textile composite, with the addition of polyurethane foam. After conducting impact tests with an energy level of 32 J, it was observed that adding material to the core improved its stiffness, increased the amount of energy absorbed, and altered the deformation mode of the sandwich structure. In the case of a simple structure, the impact resulted in the shearing of the core ligaments and the transmission of deformation to the lower face. However, the foam sample’s enhanced stiffness prevented damage to the core but instead caused the perforation of the upper-face sheet. The same positive influence on the resistance to deformation of the core was noted in [15], by adding silica gel in a bi-pyramidal sandwich structure. In addition, at the moment of upper-face puncture, the energy absorption rate of the structure filled with filler material is significantly higher than that of the base geometry. The use of vulcanized silicone rubber as a filler element in pyramid-type structures is verified in [16], leading to an increase in the core crushing strength, the upper-face puncture resistance, and the energy absorption capacity.

A brief bibliography has been compiled on the low-velocity impact loading of complex triply periodic minimal surface (TPMS) specimens, particularly those incorporating silicone filler material. Understanding how TPMS sandwich structures respond to impact loading is crucial, as it helps identify various deformation modes, such as core crushing, delamination, or damage to the outer faces. These insights are valuable for optimizing both design and material selection. Furthermore, this understanding contributes to a broader knowledge of the mechanical properties of TPMS structures, including compressive strength and ductility. Such information may lead to conclusions that support the use of these structures in applications such as structural elements in automotive and aerospace components or in protective equipment designed for superior deformation tolerance.

In summary, the growing interest in architected materials like TPMS-based cores reflects a broader shift toward optimizing structural performance through geometry-driven design. This study builds on prior work by investigating the low-speed impact performance of previously proposed TPMS core sandwich structures, both plain and filled with two-component, room-temperature vulcanized (RTV) silicone. A method for constructing impact specimens is presented, ensuring that the inserted material completely fills the volumes defined by the walls of the TPMS topologies. Through a detailed analysis of impact force, energy absorption, and failure mechanisms, the findings contribute to the understanding of how geometric configuration and material composition interact under dynamic loading. The results aim to enhance our understanding of the behavior of cellular materials with complex topologies, hollow or with filler materials, and their potential applications in structural engineering.

## 2. Materials and Methods

### 2.1. Sample Preparation

Novel TPMS structures suitable for sandwich constructions are detailed in prior research [17]. For the design of the low-velocity impact test specimens, only the two configurations that provided the best results during the compression tests highlighted in [18] were considered: the gyroid S1 and a novel TPMS design S8. Both of them used the nTopology (version 4.5.3) software for their design. Additionally, a stochastic geometry S10 was considered due to its different topology compared to the TPMS cells. S10 simulates a foam-type infill by imposing the following conditions: the struts follow the perpendicular direction between the two sandwich sheets, with an average distance between the ligaments of 3.5 mm and an average number of 6 struts intersecting at the same point.

**Figure 1:**
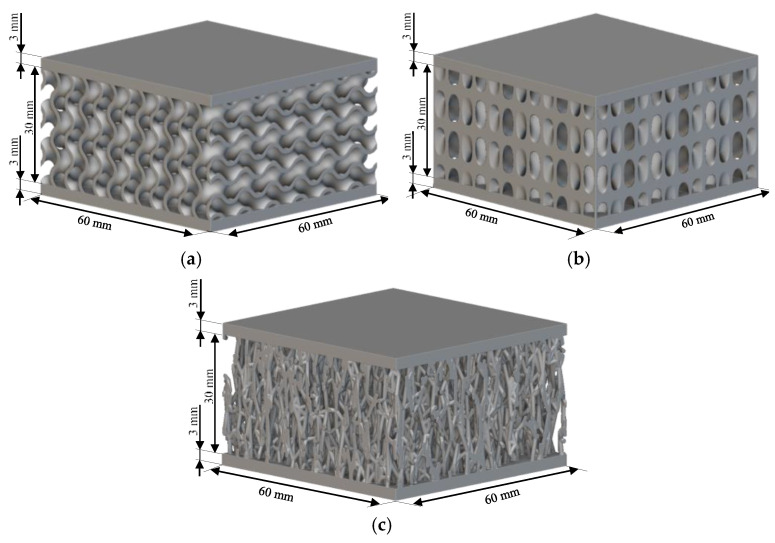
Topologies considered for low-speed impact tests: (**a**) S1; (**b**) S8; (**c**) S10.

Due to the clamping plates of the testing machine, which are designed as a large circular ring with a diameter of 60 mm, the geometry of the specimens with the dimensions specified in Figure 1 was adopted. Parts are designed using the high parameterization capacity of implicit modeling and fabricated using the stereolithography (SLA) method, from a commercial photopolymer resin, *Tough 1500 v1* (Formlabs, Somerville, MA, USA). Details on the definition and manufacturing of the samples are indicated in previous research [17]. Both the relative density of 0.3 and the dimensions of the previously presented representative volume element were maintained constant.

A total of ten specimens were printed: four identical gyroid S1 specimens, four new topology S8 specimens, and two stochastic S10 specimens. Of these, two S1 and two S8 specimens were later modified. By designing the specimens without incorporating internal closed enclosures, the entire volume defined by the constitutive walls can be filled with silicone. Below, the proposed method for fabricating these specimens is described.

In general, the addition of filler materials is accomplished by casting, without special precautions related to avoiding porosity. Although this approach is sufficient in the case of simple geometries such as the BCC [19] or re-entrant [20] topologies, when geometries with complex topologies are used, such as the proposed ones, a more efficient method is required.

Due to the complex internal volume that must be occupied by the cast material, a two-component liquid solution with a low viscosity was chosen, allowing a sufficiently long curing time for mixing, casting, and deaeration. The material characteristics of the silicone *ZA 22* (Zhermack, Rome, Italy) are presented in Table 1.

**Figure 2:**
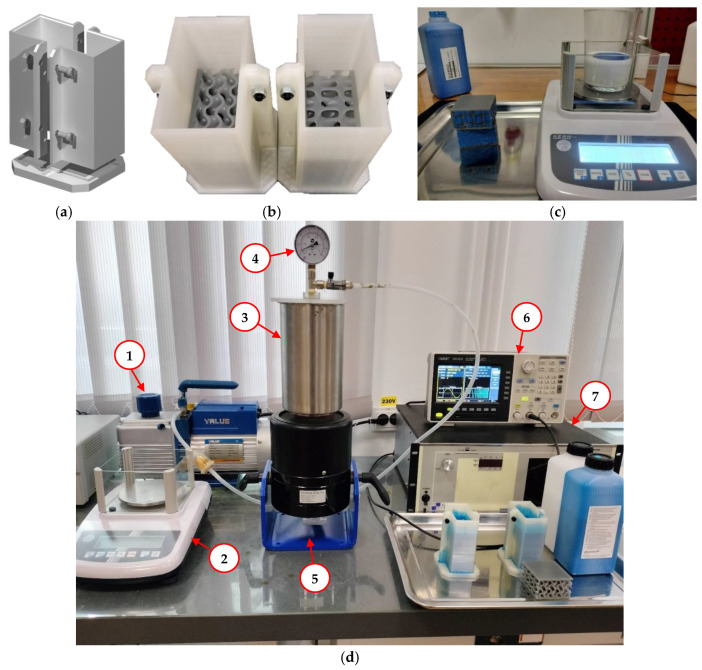
The procedure for adding silicone in the proposed samples: (**a**) Proposed mold design; (**b**) Molds with specimens prepared for filling; (**c**) Dosing and mixing of the silicone solution; (**d**) Proposed installation for deaeration of the solution.

The procedure for adding silicone in the samples is presented in Figure 2. The first step involves creating a mold using FDM additive manufacturing with semi-transparent polylactic acid, into which the filler material can be poured. To allow easy extraction after curing, a three-piece, removable solution was adopted, as shown in Figure 2a,b.

The dosage of the base solution (Figure 2c) and the catalyst was determined on the basis of equal parts by weight, by weighing on a PFB2000-2 precision balance (Kern & Sohn, Germany), followed by mixing for 60 s. Subsequently, due to the mixing process that incorporates a substantial amount of air into the silicone composition, it was necessary to deaerate the mixture. This was achieved by placing the container in an aluminum enclosure connected to a VE2100N vacuum pump (Value, China). This step was continued until no more air bubbles were observed in the solution composition, for approximately 5 min.

After mixing, the obtained composition was poured into the mold, from a height of 30 cm in a thin stream of fluid, letting the liquid gradually cover the piece to facilitate the elimination of air bubbles from its volume. Subsequently, the mold was inserted into the metal enclosure and connected to the vacuum installation. From personal observations, the short hardening times of the samples made the deaeration process insufficient. Thus, in order to obtain the best results, it was necessary to use equipment for introducing vibrations into the mass of the samples. A 2075E shaker (The Modal Shop, USA) connected to a 2050E09 power amplifier (The Modal Shop, USA) and an XDG2035 arbitrary function generator (Owon, China) were used to introduce sinusoidal vibrations with frequencies between 30 and 100 Hz and amplitudes of 30–60 mV. The proposed installation for eliminating air bubbles is represented in Figure 2d, and the relevant characteristics of the used equipment are presented in Table 2.

After curing for 24 h, the samples were removed from the mold, and the excess vulcanized silicone was eliminated by cutting. The coding of the samples, the masses, and the images of the final samples obtained after printing and adding the silicone filler are presented in Table 3. A low deviation between the masses of the samples is observed, similar to that presented in the qualitative analysis of the samples in the subsequent sections. A significant increase of over 100% is noted in the samples where silicone was used. Additionally, there is a very low deviation in the masses of similar samples, indicating a well-controlled additive manufacturing process, as well as precise silicone incorporation.

The impact analysis aims to determine whether the additional silicone influences the mechanical properties of the samples to such an extent that it provides cost-effectiveness in terms of the specific impact energy absorbed. This strategy was not applied to the stochastic geometry, S10, due to the complicated nature and uncertainty in obtaining a compact core without air voids.

### 2.2. Low-Speed Impact Test System Configuration

To analyze the mechanical behavior of the proposed sandwich panels, a series of low-velocity impact tests were performed using an INSTRON Ceast 9340 drop tower (Instron, Norwood, MA, USA), according to the ASTM D3763 standard [22]. The samples were positioned on a ring-type support with an inner diameter of 40 mm and an outer diameter of 60 mm. Selecting the 60 mm side for the samples helped ensure proper centering, which was important for guaranteeing that the impactor strikes the center of the upper face of the sandwich structure. Subsequently, they were fixed on the support using the pneumatic system of the drop tower. A hemispherical impactor with a diameter of 20 mm was used in the experiment. It was equipped with strain gauges and calibrated to allow for the measurement of the impactor’s weight as a function of time, using the DAS 64K data acquisition system. The weight of the impactor is 0.733 kg, and that of the support and the guiding mechanism is 2.5 kg. The 10 manufactured samples were subjected to impact with an imposed energy of 30 J and 40 J, respectively. To prevent multiple impacts on the sample, an anti-rebound system was implemented in cases where the upper face was not perforated. The parameters utilized during the impact tests are detailed in Table 4.

The equations accompanying impact analysis using a free-fall impact tower are simple and presented in more detail in [23].

### 2.3. Parameters Evaluated During Impact Tests

Alongside the parameters set during the testing process, several key variables and indicators were defined and considered when evaluating the results obtained. Among the parameters recorded by the impact tower, we mention the dependence between the impact force and time, which provides information about the rigidity of the structures and their structural integrity, and the value of the impactor displacement, which indicates the degree of deformation and, therefore, the ductility and strength of the samples.

The calculated impact parameters based on this data are presented below.

*Total absorbed energy* (*TAE*): defined by integrating the area under the force–displacement curve [24],

(1) $$TEA\left(\delta \right)=\int_{0}^{\delta }F\left(\delta \right)d\delta$$

*Average impact force* (*F_med_*): obtained by relating the total energy absorbed to the maximum value of the impactor displacement (*δ_max_*) [25],

(2) $$F_{med}=\frac{TAE}{\delta_{max}}$$

*Specific absorbed energy* (*SAE*): calculated by dividing the total energy absorbed by the mass of the samples [26],

(3) $$SAE=\frac{TAE}{mass}$$

*Impact force efficiency* (*IFE*): defined as the ratio between the average impact force (*F_med_*) and the maximum impact force value (*F_max_* = max|*F*(*δ*)|) [27],

(4) $$IFE=\frac{F_{med}}{F_{max}}$$

*Damping index* (*DI*): obtained by dividing the total energy absorbed by the elastic energy (*E_el_*) [28],

(5) $$DI=\frac{TAE}{E_{el}}$$

where *E_el_*—elastic energy is defined as the energy recovered by the impactor at the end of the test and calculated as the difference between the imposed impact energy *E_imp_* and the energy absorbed by each structure [27],

(6) $$E_{el}=E_{imp}-TAE$$

These parameters, together with observations related to the mode of cracking or deformation, allow the identification of shortcomings, as well as the assessment of the durability and energy absorption capacity of the structures.

## 3. Results

To compare the results obtained from the low-velocity impact tests, we graphically represented the variations in the impact force recorded by the impactor as a function of time. Using these values, we analytically determined the impactor displacement and the impact energy for a more detailed analysis of the phenomenon. Below, we present the evolution of each specimen, along with a representative figure from the tests.

### 3.1. Impact Test Results at an Impact Energy of 30 J

**Figure 3:**
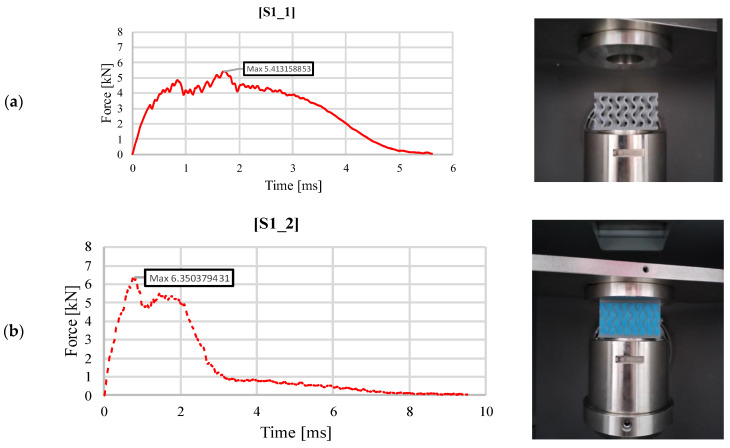
Variation of impact force as a function of time for structure S1 (30 J): (**a**) without silicone S1_1; (**b**) with silicone S1_2.

Figure 3 graphically represents the variation of the impact force as a function of time for the gyroid-type structure, without and with filler material, obtained from the impact test at 30 J. For the sandwich without silicone (Figure 3a), an initial linear increase is observed up to a local maximum value when the upper-face sheet fractures. Subsequently, a decrease in the force occurs until the core walls stiffen the response, which determines the hardening of the sample to the maximum recorded value of 5.41 kN. Unloading is carried out more slowly than loading as a result of the dissipation of the impact energy through the deformation of the sample, especially its upper face. The configuration with filler material (Figure 3b) leads to a 17% higher value of the maximum impact force. The evolution is more linear and more abrupt than in the previous case. Linearity is lost, at around 4–5 kN, when the impact force is transmitted through the upper face to the core, identifying a peak of 6.35 kN, at which point the sample cracked. The decrease in force and the next local maximum are produced, similarly, by the core failure. Subsequently, the addition of silicone, which improved the integrity of the structure, leads to a slow unloading at a much lower impact force value. Thus, a higher stiffness is identified in the case of specimen S1_2, which leads to higher impact force values, the same energy being absorbed in half the time.

**Figure 4:**
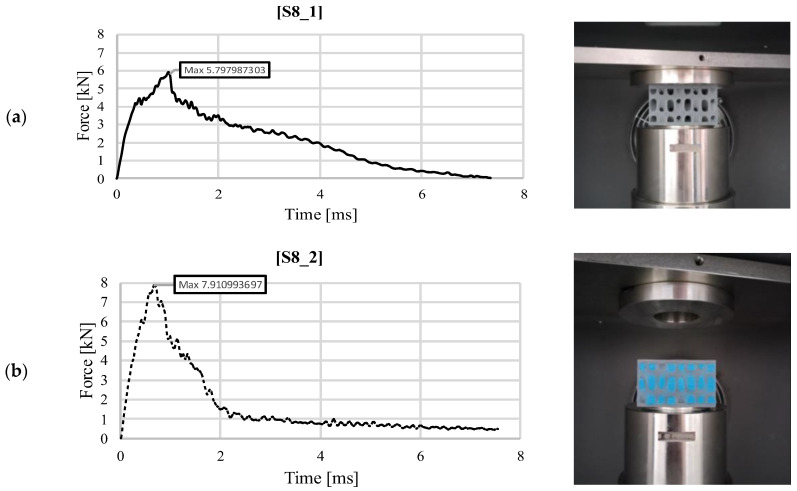
Variation of impact force as a function of time for structure S8 (30 J): (**a**) without silicone S8_1; (**b**) with silicone S8_2.

Similar behavior can be identified in Figure 4, where the same loading case is presented for the S8 topology, with and without the addition of silicone, impacted with the same energy of 30 J. Also a linear increase is distinguished, followed by force oscillations produced by the damage of the upper face up to a maximum value of 5.79 kN (Figure 4a). Here, the entire sample is damaged, the impactor continuing its movement by locally crushing the core, correlated with the slow reduction of the recorded impact force. In the case of the silicone-filled part, S8_2, the evolution is similar to the first loading case (Figure 4b). A more significant linear force increase can be identified, up to a maximum value of 7.91 kN, respectively 36% higher than the maximum recorded for the no-fill S8_1 structure. The higher stiffness provided by the filler material leads to a more obvious cracking of the sample, which makes the unloading gradient higher due to the compromised impact energy absorption capacity of the core. The slightly decreasing plateau corresponding to the impact force of 1 kN after a time of 2 ms is due to the silicone and continues until the impactor rebounds.

**Figure 5:**
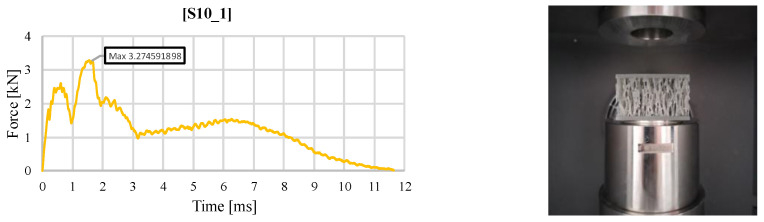
Variation of impact force as a function of time for the S10 structure (30 J).

Figure 5 represents the evolution of the impact force in the case of the sandwich structure with a stochastic core for the same impact energy of 30 J. Here, the local maxima are better delimited but different from those previously identified. If the first oscillations for the gyroid and S8 topology occurred at values between 3 and 5 kN, in the case of the S10 structure, they appear at lower values. The indentation of the upper face corresponds to the first local maximum point. A stiffening zone follows, attributed to the compression of the ligaments in the volume below the impactor. The maximum value of 3.27 kN of the impact force is reached when cracking of the upper face of the sample is produced. The following evolution is carried out over a longer period of time than in the previous cases and is due to the gradual stiffening of the core, produced by the agglomeration of the fractured ligaments in the impact zone.

**Figure 6:**
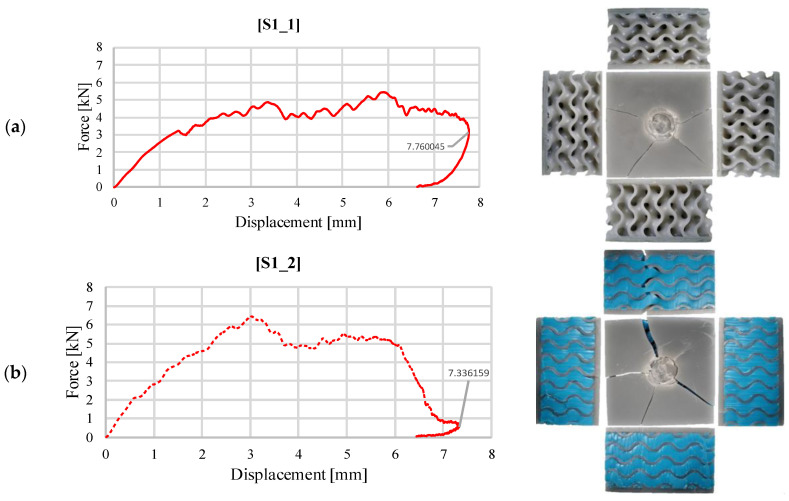
Variation of impact force as a function of displacement for structure S1 (30 J): (**a**) without silicone S1_1; (**b**) with silicone S1_2.

The following are graphical representations of the impact force as a function of the impactor displacement for the same test case. The areas under these curves represent the impact energy absorbed by the tested structures. Figure 6 shows the recorded evolutions for the gyroid-type structure, without and with silicone. In order to understand the deformation mode, each graphical representation was associated with images of the sample, taken immediately after the testing from above and from the sides. In both cases, it is observed that the faces of the samples were cracked, but they were not completely compromised, the impactor rebounding from the samples and being caught by the anti-rebound system, in order to avoid multiple impacts. The residual deformation at the end of the test signifies the maximum penetration depth of the impactor into the body of the sandwich specimen and was verified by measuring it with a caliper, immediately after testing, the values being the same as those indicated on the graph. Comparing the two configurations, S1_1 and S1_2, there are no substantial differences in terms of maximum impactor displacement. The fracture of the structure with filler material is more visible (Figure 6b) due to the higher stiffness and impact force. The effect of the filler material is also visible, in the loop made at the end of the curve, where due to the elasticity of the silicone, the deformation increases rapidly, but the energy absorbed in this area is insignificant.

**Figure 7:**
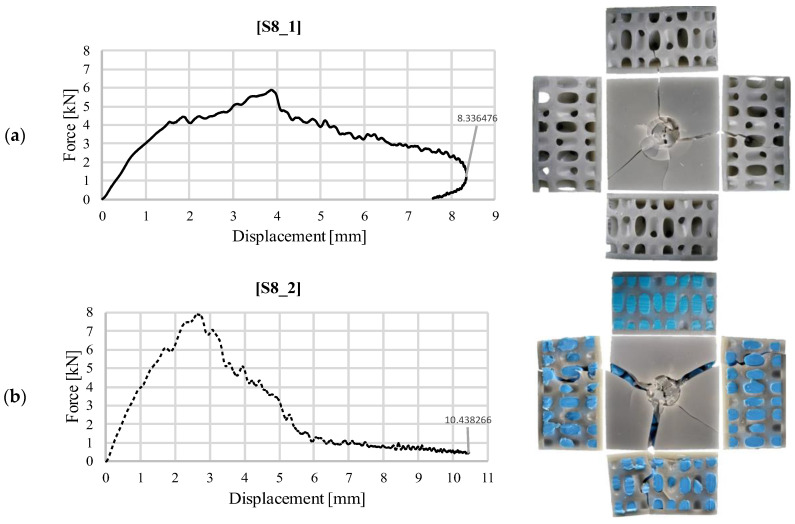
Variation of impact force as a function of displacement for structure S8 (30 J): (**a**) without silicone S8_1; (**b**) with silicone S8_2.

Figure 7 shows the same graphical representations for the S8 structure. A similar evolution is observed in the case of the simple structure, with a slightly larger penetration into the volume of the part than in the case of the gyroid. When silicone filler was used, the deformation of the upper face was accompanied by cracking of the entire sample. This is also visible in the images taken after the tests but also in the graphical representation in Figure 7b, where the displacement of the impactor over the 6–10 mm portion at a low force value is produced by the compression of the silicone. In addition to the role of increasing the rigidity of the structure, the added silicone also helped to avoid the complete fracture of the sample, holding the internal walls together.

**Figure 8:**
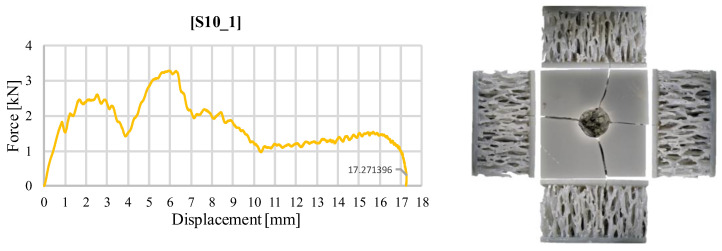
Variation of impact force as a function of displacement for structure S10_1 (30 J).

The different deformation encountered in the stochastic topology S10 is visible in Figure 8. It is observed that the cracking of the upper face did not propagate into the core of the sample, which was encountered in the other structures. Instead, the impactor had a much larger displacement, approximately equal to half the height of the sandwich structure, by fracturing the thin ligaments, which, captured under the impactor, led to a slow stiffening until the predefined impact energy was reached.

**Figure 9:**
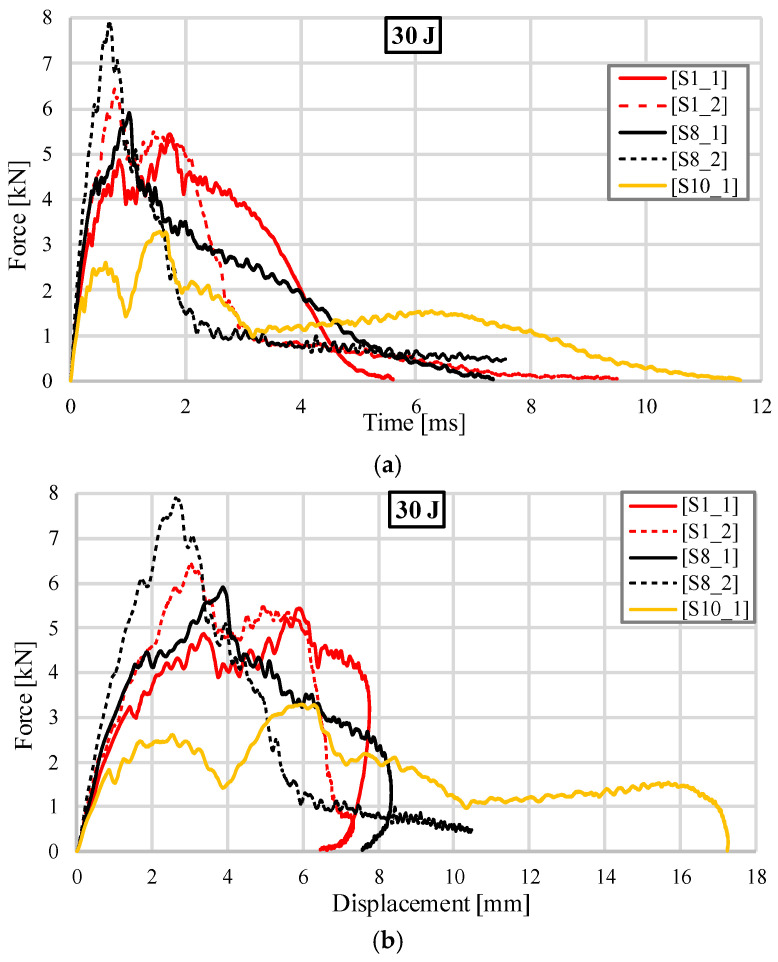

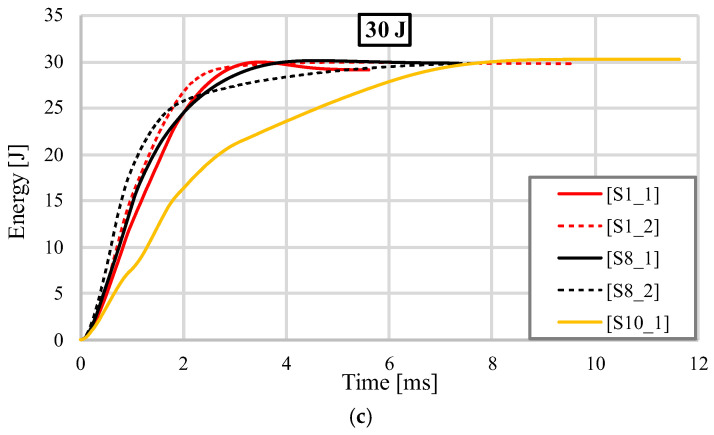
Comparison of impact results (30 J): (**a**) Impact force versus time; (**b**) Impact force versus displacement; (**c**) Impact energy versus time.

Figure 9 illustrates the comparison of all samples subjected to low-velocity impact with an energy of 30 J. In Figure 9a, the evolution of the impact force as a function of time is presented. The solid lines represent the unfilled samples, while the dotted lines correspond to the samples filled with silicone. When comparing the S1 geometry to the S8 geometry without silicone, it is observed that the S8 exhibits higher maximum impact force values and greater stiffness in the initial region. This finding aligns with the results of previous compression tests conducted [18]. Although both samples suffered crack propagation from the upper face into the core volume, the unloading of the S8_1 specimen starts earlier and is carried out more slowly as a result of the greater deformation in the central area. The shape of the yellow curve of the stochastic structure differs from the TPMS geometries due to the distinct deformation mechanism. The reduced transmission of stresses into the core and the localization of deformation in the impact area, caused by the crushing of the ligaments, are key manifestations of this phenomenon. This crushing results in an increase in the impact force at high levels of impactor displacement. Specimens filled with silicone exhibit higher stiffness and more distinct peaks in impact force for both configurations. However, there are notable differences: the gyroid structure S1 exhibited a decrease in force in two phases, first due to the crushing of the upper face and then due to cracking. In contrast, the S8 sandwich configuration failed much more rapidly, experiencing significant fractures that resulted in a steeper reduction in force. The overlap of the two dotted curves corresponding to 1 kN force, determined by the compression of the silicone after the integrity of the samples was compromised, can also be observed. Comparing the two types of geometries with their silicone-filled counterpart, S1_1 and S1_2, and S8_1 and S8_2, respectively, we can establish similar behavior. In both cases, the addition of silicone led to an increase in the maximum impact force and increased the rigidity of the structure, with energy absorption occurring in a shorter time interval.

Figure 9b suggests the evolution of the impact force as a function of displacement. No significant differences in the deformation values in the impactor area are observed between the silicone-free structures, S1_1 and S8_1, or between the two variants of the gyroid-type structure, S1_1 and S1_2. Different behaviors were observed for topology S8_2, where the sample cracked in multiple directions down to the lower surface of the structure. In contrast, topology S10_1 exhibited a local indentation that was twice as deep compared to the other samples. In both of these cases, the impactor remained fixed within the sample and did not rebound due to the significant deformations it caused.

Figure 9c shows the evolution of the impact energy as a function of time. It is noted that all the samples reached the specified impact energy, but at different times. Although they initially have a higher growth rate, the dotted curves corresponding to the silicone-filled solutions reach the maximum energy value later due to the large cracks and the energy take-up towards the end of the impact only by the silicone, which happens with a much lower rate. The stochastic geometry has a distinct evolution, through slower growth over the entire time domain.

### 3.2. Impact Test Results at an Impact Energy of 40 J

**Figure 10:**
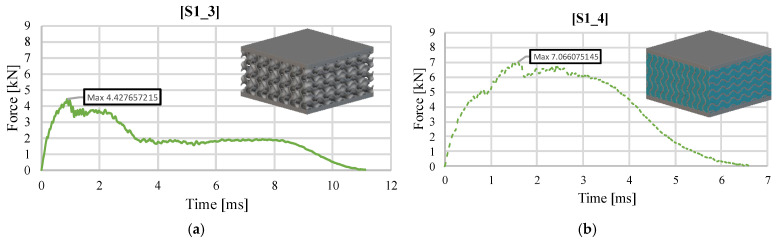

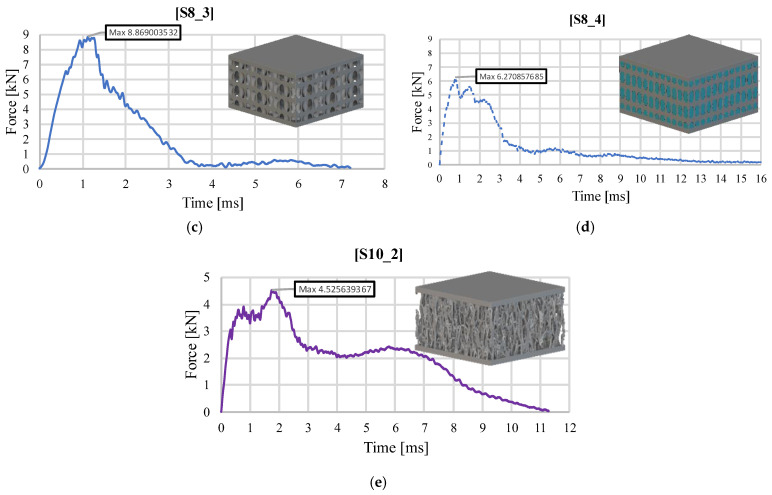
Variation of impact force as a function of time (40 J) for (**a**) S1_3 without silicone, (**b**) S1_4 with silicone, (**c**) S8_3 without silicone, (**d**) S8_4 with silicone, and (**e**) S10_2 without silicone.

In previous tests, it was found that at lower impact energy, the damage caused is generally localized at the upper surface and the area around the impactor but can also lead to cracking of the samples. Next, the impact tests performed with an energy of 40 J are presented, on the same types of geometries, to verify whether the failure mode changes. Figure 10 shows the graphical representations of the impact force as a function of time for the proposed geometries, with and without filler material.

The unfilled gyroid S1_3 shows behavior similar to that of the 30 J load case, in Figure 3a, with the observation that it takes place over a longer period of time due to the more pronounced deformation in the impact area, which causes the impactor to remain stuck in the sample. The maximum impact force is lower than for the 30 J impact, with a maximum value of 4.42 kN (Figure 10a). After the local deformation of the upper face, the force decreases, followed by a short plateau until its total cracking. Subsequently, the core of the sample is deformed, and a stiffening is observed in a short time interval, based on the yielding and agglomeration of the material in the impactor area. The S8 solution without silicone, S8_3, has behavior almost identical to that identified following the 30 J tests. A linear loading zone up to a value of 4–5 kN specific to the plastic deformation of the upper face is observed, followed by a subsequent stiffening produced by the transmission of the stress to the core of the sample. The maximum force recorded is 8.87 kN. Immediately after this, cracks are observed propagating along the entire height of the structure, leading to a rapid decrease in the impact force (Figure 10c). This is achieved with a higher gradient than in the case of the 30 J impact but in a similar time. Comparing the two specimens with each other, S1_3 and S8_3, it is found that the maximum impact force increases by up to 100% in favor of the S8 topology.

Regarding the specimens filled with silicone, the S1_4 specimen had different behavior. It did not deform as much as the hollow S1_3 configuration, with filler material helping to prevent the core from cracking and to record a maximum force value 60% higher, respectively, 7.06 kN (Figure 10b). The lack of complete failure is also observed in a much shorter time, in which the impact phenomenon occurs, of approximately 7 ms. The filled S8_4 specimen, compared to the S8_3 counterpart, highlights behavior that is not consistent with the observations valid for the other specimens. If, in the other cases, the addition of silicone led to higher impact force values compared to the hollow solution, in this case, the maximum value for S8_4 is 29% lower than S8_3, namely, 6.27 kN (Figure 10d). Similar behavior is observed with S8_2 (Figure 4b). After reaching the maximum force value, the rapid unloading triggered by the fracture of the sample core caused the last portion of the impact energy to be absorbed solely by the silicone. The stochastic structure S10_2 follows the same pattern as the one in Figure 5. Both cases highlight an initial local maxima of the impact force, specific to the initiation of the deformation of the upper face, followed by the transmission of the energy to the ligaments in the impact area, resulting in a global maximum 38% higher, and the subsequent rapid unloading produced by the ligaments fracture, until their agglomeration in the contact area and the stiffening (at a force above 2 kN) of the sample are visible.

**Figure 11:**
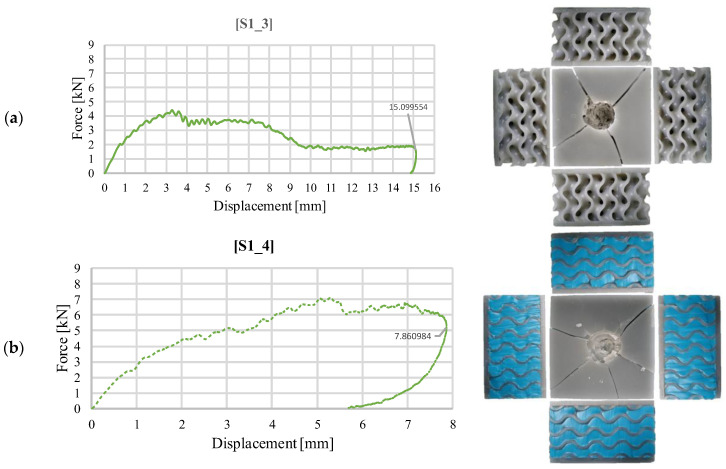
Variation of impact force as a function of displacement for structure S1 (40 J): (**a**) without silicone S1_3; (**b**) with silicone S1_4.

Figure 11 presents a graphical representation of the impact force as a function of the impactor displacement for structures with a gyroid core. Unlike the similar failure modes observed during the impact tests with an energy level of 30 J (as shown in Figure 6), the sample without silicone exhibits different behavior at a higher impact energy. The cracking of the upper face also extends to the core, although it does not crack along its entire length. Additionally, the increased energy results in the crushing of the walls in the contact area and even its penetration (Figure 11a), leading to a high deformation of 15.1 mm, similar to that seen in the stochastic solution, where failure is primarily localized in the impact area. The addition of silicone enhances the stiffness of the structure, resulting in the upper face of sample S1_4 not being penetrated (Figure 11b). Consequently, the values of the impactor displacement are significantly lower, with a maximum value of 8.86 mm, which decreases towards the end of the impact event. This response resembles the one observed in the initial test, with the key difference being that the fracturing of the sample does not occur throughout the entire height of the core. Furthermore, the loop indicated in Figure 6b, which reflects the effect of silicone, is not present.

**Figure 12:**
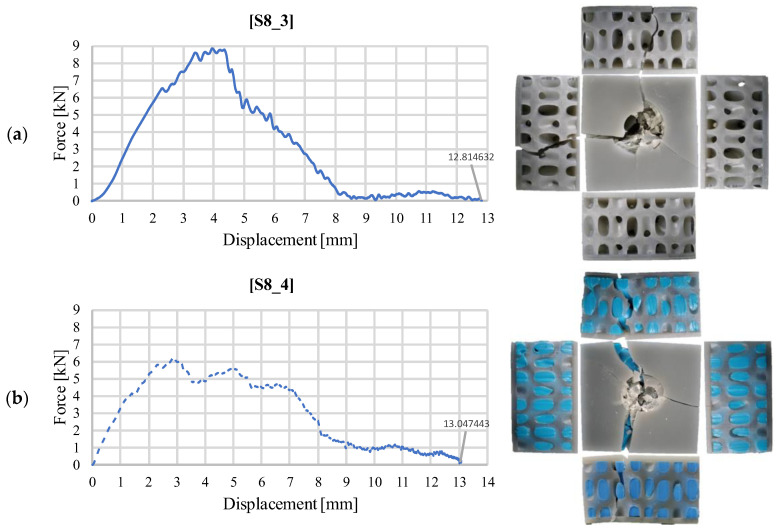
Variation of impact force as a function of displacement for structure S8 (40 J): (**a**) without silicone S8_3; (**b**) with silicone S8_4.

Figure 12 shows the curves specific to the S8 structure. The S8_3 sample has a much stiffer response to the 40 J impact than the 30 J test (Figure 12a). However, unlike the first test, where the unfilled sample did not break (Figure 7a) but the impactor rebounded, in this test, the integrity of the sample was compromised in both configurations. In the case of S8_4, the behavior is similar, with the sample being completely cracked. The two local maxima specific to the face sheet failure and core failure can be observed (Figure 12b), as well as the compression of the silicone over the last 5 mm, at a force value similar to that in the previous observations. The deformation is severe, as the impactor penetrates the sandwich structures with about 13 mm in both cases.

**Figure 13:**
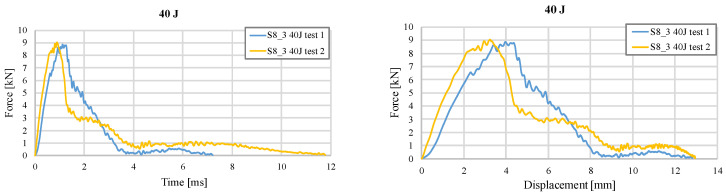
Variation of impact force as a function of time and displacement for unfilled structure S8_3 (40 J) in two successive tests.

The unusual response of the S8_3 sample, where the impact force of the unfilled structure exceeds that of the silicone-filled version, was confirmed in a follow-up test, as shown in Figure 13, to ensure the repeatability of the results. A relatively small difference is noted between the two tests, with comparable values for the maximum impact force and similar displacements of the impactor at the point of total failure of the sample.

**Figure 14:**
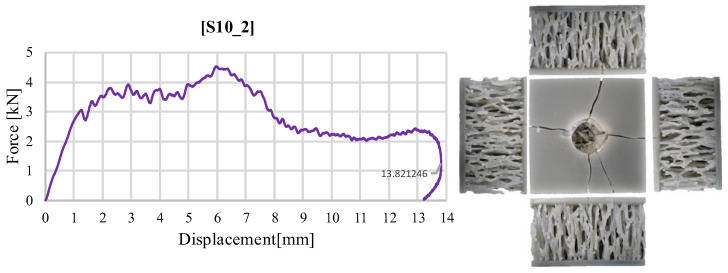
Variation of impact force as a function of displacement for structure S10 (40 J).

The force–time diagram for the sandwich structure with a stochastic core at an impact with 40 J shows a pattern similar to that observed in the first test. As illustrated in Figure 14, a failure mode comparable to the initial test is evident, characterized by cracking of the face and indentation of the impactor into the stochastic core until it becomes blocked. This effect, caused by the accumulation of torn ligaments beneath the impactor, is noticeable at the end of the graphical representation of force evolution. The primary difference is that the deformation recorded in this test was slightly less than that seen in the first test (as shown in Figure 8), with maximum values approaching 14 mm. However, the impact force recorded during this test was higher, and the overall pattern remained consistent.

**Figure 15:**
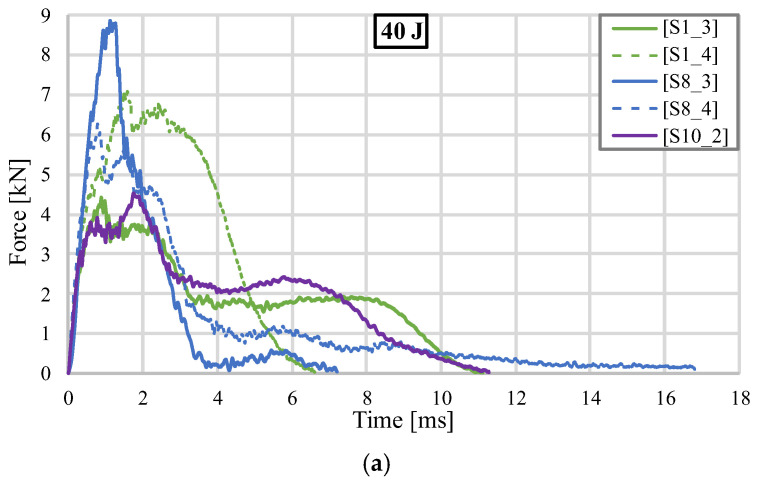

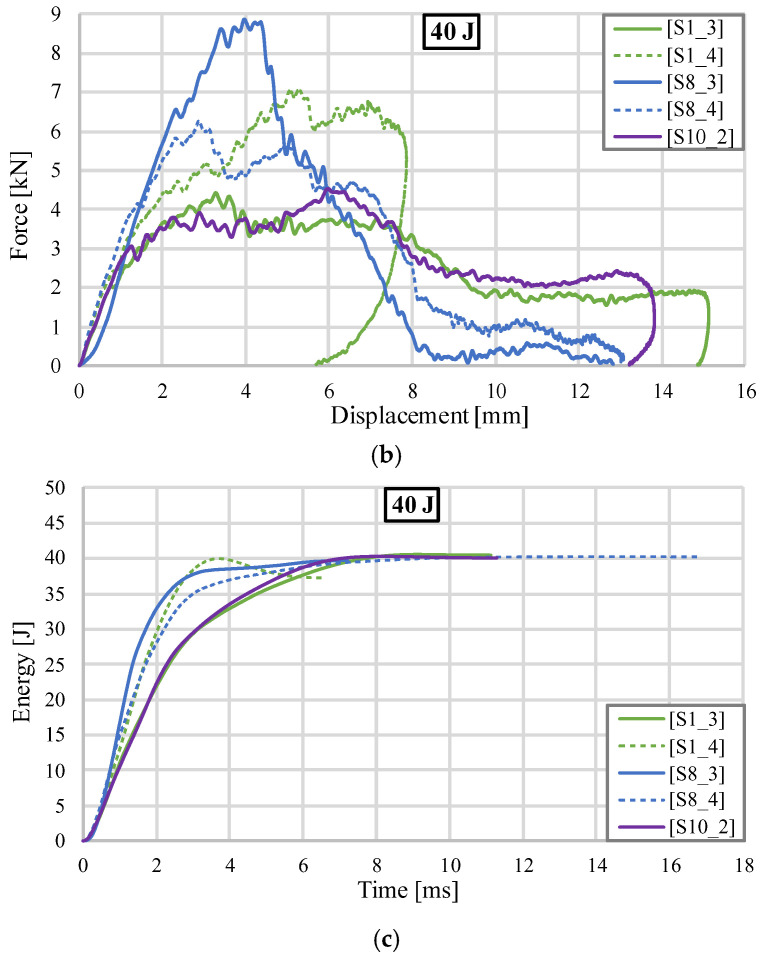
Comparison of impact results (40 J): (**a**) Variation of impact force as a function of time; (**b**) Variation of impact force as a function of displacement; (**c**) Variation of impact energy as a function of time.

Figure 15 shows the comparison of all five samples subjected to impact with an energy of 40 J. Figure 15a shows the impact force as a function of time. Comparing the continuous curves specific to the hollow samples, it is observed that the gyroid structure presented a similar evolution to that recorded in the first test but spread over a much wider period of time. As already indicated, S8_3 has an atypical response, with a much higher stiffness, the force value suggesting that a different impact speed or energy can induce a different type of response in TPMS-type geometries. In the case of the dotted curves specific to the solutions with additive material, S1_4 shows an improvement in performance, in terms of both maximum impact force and time in which the phenomenon occurs, compared to its counterpart without silicone. However, the filled S8_4 presents lower force values than the unfilled S8_3 structure, the rapid cracking leading to a higher portion of the energy being absorbed by the filler material after the structural integrity of the specimen was lost.

Figure 15b shows that the gyroid core structure, S1_3, approaches behavior similar to the stochastic geometry, S10_2, when tested at higher impact energies, and the local crushing of the walls approximates the response produced by ligament fracturing. Considering the objective of achieving the lowest possible deformation values, it was observed that the gyroid structure with silicone filler, S1_4, exhibited the best performance. It only experienced superficial fractures on the upper surface, and the impactor rebound was evident in the displacement value on the graph at the end of the test.

In terms of how the impact energy is absorbed, all tested specimens successfully reached the targeted energy value of 40 J during the experiments, displaying trends similar to those shown in Figure 12. The hollow gyroid structure, labeled S1_3, exhibited behavior similar to that of the stochastic sample. This can be attributed to the arrangement of the walls, which allowed for significant local deformations without compromising the entire sample’s integrity. On the other hand, the silicone-filled gyroid, S1_4, demonstrated effects similar to the first test. This led to an increase in the stiffness of the sample and facilitated quicker energy absorption with minimal deformations. The green dotted curve in Figure 15c highlights that its maximum point indicates the imposed impact energy during the test, while the total energy absorbed by the sample is represented by the value at the end of the test. The difference between these two values is referred to as the recovered elastic energy and is observed in tests where the samples do not suffer complete failure [29]. In contrast, the hollow topology S8_3 exhibited an unexpected response due to its high stiffness; however, this was insufficient to meet the required impact energy threshold. This threshold was only achieved after the structural integrity of the part was compromised, with the crushed walls in the central area absorbing the remaining 5 J of energy.

## 4. Discussion and Conclusions

This research aims to expand the understanding of the behavior of TPMS and stochastic structures proposed in previous research through low-velocity impact tests. The two most promising topologies and the stochastic topology were chosen. As an extension to the study, given the existing interest in this field in the specialized literature, filling the empty volume in the TPMS cores with a two-component silicone solution, vulcanized at room temperature (RTV silicone), was proposed. This approach is feasible because the considered topologies have one or more continuously connected volumes that can be filled without forming closed enclosures. Specific recommendations were implemented to ensure the homogenization of the silicone material. Additionally, a method was proposed to eliminate core porosity within the printed samples. Based on trial-and-error observations, it was found that using a vacuum pump alone is insufficient to achieve a uniform structure. The process involves mixing the silicone solution and then using a vacuum pump to extract the air that becomes trapped during mixing. Once mixed, the solution is poured into molds designed for easy extraction. The molds are then placed on a shaker to introduce vibrations, and the vacuum pump is connected again for 8 to 10 min. After 24 h, the samples are removed from the molds, and any excess material is trimmed away. The resulting samples were tested for low-velocity impacts with energy levels of 30 J and 40 J. To enable a comparison of the performance of the proposed structures, the parameters shown in Table 5 were evaluated. The highest and lowest values for each test set are highlighted in green and orange, respectively.

A key observation is that the manufacturing process remains optimal, as indicated by the minimal deviations in mass among specimens of the same type. The standard deviation for samples without filler material was 0.66, while for those with added silicone, it was 0.49. However, filling the sandwich-type specimens results in a final mass that is over 219% higher than the initial mass, which could be a disadvantage in situations where weight is a critical factor. When examining the maximum force achieved, it is noted that the use of silicone resulted in stiffer samples in three out of four cases, leading to higher impact forces. However, the maximum force was recorded for the unfilled S8_3 structure tested at an impact energy of 40 J, where the addition of silicone surprisingly yielded a lower force value. To address this unexpected outcome, an additional test was conducted to assess the repeatability of the data, which confirmed the results. Additionally, it was observed that the S8-type structure exhibited higher impact force values compared to the classic S1 gyroid structure. In contrast, the sample with a stochastic core recorded the lowest values during both tests, attributed to the reduced stiffness of the ligaments used.

Most of the time, a high impact force was also accompanied by a high value of the impactor displacement and, implicitly, of the sample indentation. This is the case for the S1_2, S8_2, S8_3, and S8_4 structures, where cracking of the upper face also caused the core to fracture over the entire height. So, although the S8 topology presents higher values for the maximum impact force, it is more susceptible to the loss of structural integrity, especially at high energy values. In contrast, samples S1_1, S8_1, and S1_4 showed only damage to the upper face, with minimal cracking deep into the core. Structures S10_1 and S10_2 allowed high local deformations, where the impactor destroyed the ligaments in the impact area, without generating total destruction of the parts. This phenomenon is most visible in the stochastic topology but was also encountered in the 40 J energy test on the gyroid sample, S1_3.

During the tests, all samples met the specified thresholds of 30 J and 40 J. However, some samples fully absorbed this energy, while others reflected a small portion back to the impactor. The amount of absorbed energy is influenced by the plastic damage of the samples. Sample S1_3 demonstrated the best absorption capacity, deforming up to 15 mm. In contrast, sample S1_4 absorbed the least impact energy, returning 2.83 J to the impactor due to its higher stiffness. It can be concluded that a structure with a low level of energy reintroduced into the system at the end of the test, as shown in the penultimate column of Table 5, is advantageous for energy absorption applications. This characteristic can help minimize damage to the object that impacts the sample. Conversely, if the goal is to ensure that the struck object sustains minimal damage, higher values of recovered energy are desirable, as seen in gyroid-type samples.

Although the S8 topology presented in most cases higher values of impact force, the significant deformations cause the average impact force to have the highest value in the case of sample S1_4, as 4.73 kN, due to the lack of plastic deformation and impactor rebound. At the opposite pole are the topologies with a stochastic core that presented the most significant deformations, resulting in a lower average impact force. If we take into account the additional mass introduced by the silicone filler, it is observed that the hybrid structures lead to much lower specific absorbed energy values. Values up to 215% lower were obtained following tests with an impact energy of 30 J, respectively, 240% for 40 J.

The significance of oscillations in the impact force value is emphasized by its efficiency. A narrower range of these oscillations indicates that the structure can absorb impact energy more effectively, minimizing large stress gradients during the event, which can lead to substantial plastic deformations. In this context, structures with gyroid and stochastic cores demonstrate efficiencies ranging from 0.6 to 0.7.

The damping index reflects the system’s ability to reduce vibrations following an impact. A lower damping index typically corresponds to higher vibrations and rebound phenomena from the impactor. Conversely, structures with higher damping index values are better at dissipating energy and attenuating vibrations more quickly. In most cases, nearly all the absorbed energy is used to penetrate the samples, transferring only a small amount of energy back to the impactor, resulting in very high damping. The exceptions to this trend are found in structures S1_1 and S1_4.

Taking all this into account, it can be concluded that sandwich structures with TPMS and stochastic cores are optimal solutions for applications where energy absorption is important. Structure S8 showed higher performance at lower impact energy values, but it deformed plastically faster by losing structural integrity at higher energies. At the same time, the stochastic structure presented the most predictable response, localizing the deformation, without endangering the integrity of the panel, in accordance with similar observations in the literature [7].

When using filler material in structural applications, this approach generally leads to a stiffer response in the structures, particularly when subjected to high impact forces. However, it does not significantly improve the energy absorption properties. Given the complexities of producing such homogeneous structures on a large scale and the additional weight they add, this method is practical only for specific applications, where the additional mass is not a limiting factor. Here, the primary goal was to protect the sandwich panels, silicone being used as a binder between the chambers created in the core volume. This applies, however, only for topologies that create continuous volumes inside the sample, so that the elastic filler material can act as a binder for the entire configuration. The suggestion to employ a bi-component silicone filler serves as an example to illustrate the hybrid concept that could benefit from a TPMS-type geometry and its manufacturing feasibility. These proposed structures have potential applications in various fields, including vibration damping, impact mitigation systems, and other specialized areas.

In summary, the investigation confirms that TPMS and stochastic core sandwich configurations exhibit distinct mechanical responses under low-velocity impact loading, shaped by their geometry and the presence of filler materials. While TPMS topologies, particularly S8, show enhanced stiffness and peak force resistance, their susceptibility to structural failure at higher energies presents a trade-off. Stochastic cores, by contrast, offer a more controlled and predictable failure mode. The addition of RTV silicone improves stiffness and damping properties but introduces significant additional mass, limiting its practical utility in weight-sensitive applications. These insights contribute to the broader understanding of cellular structural performance and highlight key design considerations for tailoring sandwich composites to specific functional requirements in engineering applications.

## Figures and Tables

**Table 1:** Properties of the bi-component material ZA 22 used to fill the samples [21].

Characteristic	Value
Mixing ratio (parts by weight)	1:1
Viscosity after mixing	4500 ± 300 cP
Density after mixing	1.13 g/cm^3^
Mixing time at 23 °C	1 min
Application time at 23 °C	14–17 min
Curing time at 23 °C	60–90 min
Hardness–Shore A after 24 h	21 ± 2 shA
Tensile strength after 24 h	4 ± 0.2 N/mm^2^
Specific strain at break after 24 h	480%
Reproduction details	Up to 2 µm
Dimensional variation	max 0.05% after 24 h

**Table 2:** Main characteristics of the equipment from Figure 1 used to fill with silicone.

No. in Figure 2	Equipment	Characteristics
1	Vacuum Pump VE2100N	Flow rate: 283 l/min; Vacuum depth: 2 × 10^6^ bar
2	Precision Balance PFB2000-2	Accuracy: 0.01 g
3	Vacuum Chamber	Made of aluminum
4	Manometer	Measuring range: −1~0 bar relative pressure
5	Shaker 2075E	Maximum stroke: 25.4 mm; Maximum weight: 3.2 kg; Maximum speed: 1.8 m/s
6	Arbitrary Function Generator XDG2035	Frequency: 1 µHz~15 MHz; Accuracy: ±2 ppm
7	Power Amplifier 2050E09	Output voltage: 50 V; Output power: 1000 VA

**Table 3:** Properties of the bi-component material ZA 22 used to fill the samples.

Sample	Figure	Mass [g]	Sample	Figure	Mass [g]
S1_1	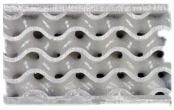	66.99	S8_1	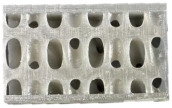	66.61
S1_2	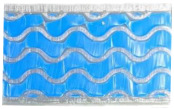	67.47	S8_2	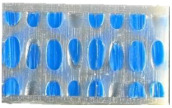	65.87
		147.2 with silicone			146.3 with silicone
S1_3	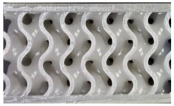	66.07	S8_3	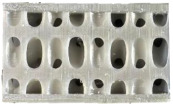	66.01
S1_4	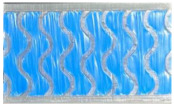	66.9	S8_4	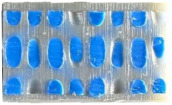	66.6
		146.5 with silicon			147.3 with silicon
S10_1	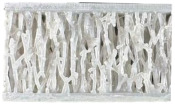	67.77	S10_2	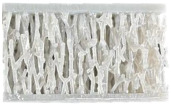	66.34

**Table 4:** Parameters used during low-speed impact tests.

Impact Energy	Fall Height	Falling Mass	Impact Velocity	Samples Tested
30 J	946 mm	3.233 kg	4.31 m/s	S1-1, S1-2, S8-1, S8-2, S10-1
40 J	779 mm	5.233 kg	3.91 m/s	S1-3, S1-4, S8-3, S8-4, S10-2

**Table 5:** Parameters for evaluating the performance of structures under impact loads.

Sample	Impact Energy [J]	Mass [g]	Maximum Force [kN]	Maximum Deformation [mm]	Total Absorbed Energy [J]	Average Impact Force [kN]	Specific Absorbed Energy [J/kg]	Impact Force Efficiency [-]	Recovered Energy [J]	Damping Index [-]
**S1_1**	30	67	5.413	7.76	29.07	3.75	433.95	0.692	0.93	31.25
**S1_2**	30	147	6.35	7.33	29.72	4.05	201.90	0.639	0.28	106.14
**S8_1**	30	66.6	5.798	8.33	29.78	3.58	447.08	0.617	0.22	135.36
**S8_2**	30	146	7.911	10.43	29.88	2.86	204.24	0.362	0.12	249
**S10_1**	30	67.8	3.275	17.27	30.19	1.75	445.48	0.534	0.19	158.89
**S1_3**	40	66.1	4.428	15.09	40.35	2.67	610.72	0.604	0.35	115.28
**S1_4**	40	147	7.067	7.86	37.17	4.73	253.72	0.669	2.83	13.13
**S8_3**	40	66	8.869	12.81	39.75	3.10	602.18	0.350	0.25	159
**S8_4**	40	147	6.271	13.04	39.61	3.04	268.91	0.484	0.39	101.56
**S10_2**	40	66.3	4.526	13.82	39.93	2.89	601.90	0.638	0.07	570.42

## Data Availability

The raw data supporting the conclusions of this article will be made available by the authors on request.
